# P-1576. An Exploration of the Utility of Urinalysis Criteria in Predicting Urine Culture Positivity

**DOI:** 10.1093/ofid/ofae631.1743

**Published:** 2025-01-29

**Authors:** Shivum Patel, J Njeri Wainaina

**Affiliations:** Medical College of Wisconsin, Milwaukee, Wisconsin; Medical College of Wisconsin, Milwaukee, Wisconsin

## Abstract

**Background:**

Urinalysis (UA) testing with reflex to culture is commonly ordered and is considered a tool for both diagnostic and antibiotic stewardship. Criteria for culture delineate normal from abnormal values leading to use for diagnosis of urinary tract infections (UTI). However, urinalysis criteria which predict urine culture growth that correlate with UTI are not well elucidated. The objective of this study is to evaluate the utility of several different indicators in urinalysis as reflex criteria to urine culture.

**Figure:**
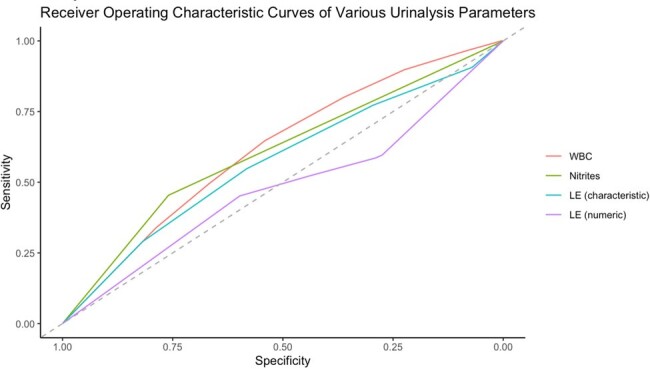

The area under the curves are: 0.618 for WBCs, 0.607 for nitrites, 0.570 for LE by semi-quantitative testing, and 0.479 for LE by numeric testing.

**Methods:**

Retrospective review of 14,212 unique de-identified samples of paired urinalysis and urine culture collected in the Froedtert Health System in Milwaukee, Wisconsin from 9/22/22 to 8/23/23. All urine specimens were included regardless of collection method. Cultures were deemed to be positive if growing at least 100,000 colony-forming units (CFU) of a likely urinary pathogen and were evaluated for white blood cells (WBC), leukocyte esterase (LE), and nitrite positivity in the corresponding urinalysis.

**Figure:**
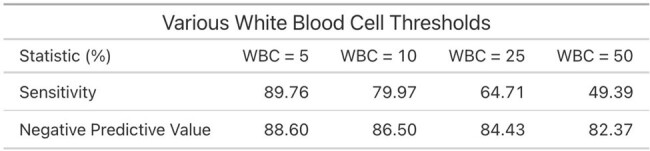

**Results:**

For each criterion, sensitivity and negative predictive values (NPV) were calculated at varying thresholds. A threshold of 5 WBCs provided the greatest NPV at 88.60%, and the greatest sensitivity at 89.76%. Next, receiver operating characteristic (ROC) curves were generated and the area under the curves were: 0.618 for WBCs, 0.607 for nitrites, 0.570 for LE by semi-quantitative testing, and 0.479 for LE by numeric testing.

**Figure:**
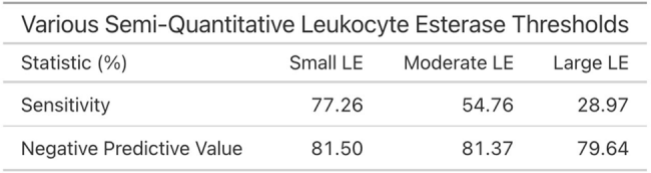

**Conclusion:**

Of the three urinalysis criteria, WBC had the best sensitivity and NPV but the ROC curves suggest that none of the tests predict significant urine culture growth well, emphasizing the importance of clinical context in both determining when to test and how to interpret. As diagnosis of a UTI does not simply rest on certain CFU growth or uropathogen on urine culture, but requires incorporation of history and physical findings, the next stage will be to correlate urine testing results with symptoms.

**Figure:**
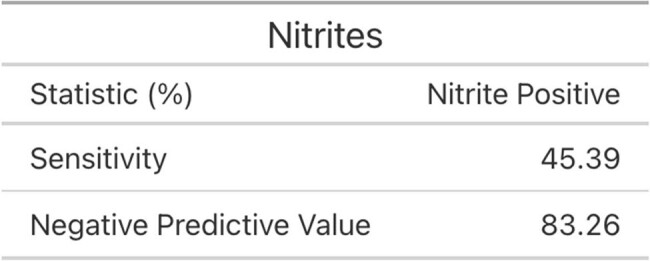

**Disclosures:**

**All Authors**: No reported disclosures

